# HELIOS Action: Advancing research, education, and equity in hemoglobinopathies across Europe and beyond

**DOI:** 10.1002/hem3.70258

**Published:** 2025-12-08

**Authors:** Sotiroula Chatzimatthaiou, Fedele Bonifazi, Anna C. Gimbert, Raffaella Colombatti, Francesco Cremonesi, Andreas Glenthøj, Elisabetta Mezzalira, Coralea Stephanou, Jan Traeger‐Synodinos, Darko Antic, Burak Durmaz, Eleni Gavriilaki, Baba Inusa, Annalisa Landi, Mariangela Pellegrini, Francesca Basile, Stephanie Muth, Holger Cario, Eleni Katsantoni, Dilem Ruhluel, Carsten Werner Lederer, María del Mar Mañú‐Pereira, Petros Kountouris

**Affiliations:** ^1^ Molecular Genetics Thalassaemia Department The Cyprus Institute of Neurology & Genetics Nicosia Cyprus; ^2^ Fondazione per la ricerca farmacologica Gianni Benzi onlus Bari Italy; ^3^ Rare Anemia Disorders Research Laboratory, Group of Translational Research in Cancer and Blood Disorders in Children Vall d'Hebron Institut de Recerca Barcelona Spain; ^4^ Department of Women's and Children's Health University of Padua Padua Italy; ^5^ EPIONE Research Group INRIA de l'Université de la Côte d'Azur Valbonne France; ^6^ Danish Red Blood Cell Center, Department of Hematology Copenhagen University Hospital – Rigshospitalet Copenhagen Denmark; ^7^ National and Kapodistrian University of Athens Athens Greece; ^8^ Clinic for Hematology, University Clinical Center of Serbia, Faculty of Medicine University of Belgrade Belgrade Serbia; ^9^ Department of Medical Genetics Ege University, Faculty of Medicine Bornova Izmir Turkey; ^10^ Aristotle University of Thessaloniki Thessaloniki Greece; ^11^ Faculty of Life Sciences and Medicine King's College London London United Kingdom; ^12^ Medical and Science, Rare Disease, Novo Nordisk, Novo Alle 1 Bagsvaerd Denmark; ^13^ Département d'Hématologie et Immunologie, Hôpital St. Louis, Assistance Publique Hôpitaux de Paris Hôpital Saint‐Louis Paris France; ^14^ StoryRelator Ltd. London United Kingdom; ^15^ Department of Pediatrics and Adolescent Medicine and Center for Rare Hematopoietic Disorders and Immunodeficiencies (ZSHI Ulm) Ulm University Medical Center Ulm Germany; ^16^ Basic Research Center, Biomedical Research Foundation Academy of Athens Athens Greece

Recent decades have witnessed remarkable advances in hematology research,^1^ and yet, for many affected populations, particularly those with sickle cell disease (SCD) and Thalassemia, more so in low‐resource settings, clinical outcomes remain suboptimal.^2^ Despite being among the most common inherited disorders globally, with an estimated 330,000 conceptions annually,^3^ patients with hemoglobinopathies encounter fragmented care systems and unequal access to diagnosis and treatment, and there is limited coordination in research and clinical practices across countries.^4^

Although hemoglobinopathies have gained recognition as a global public health concern,^3^ there are still four persistent challenges. First, there are only a limited number of harmonized, multicentric studies that capture the genetic and clinical variability of these disorders to enable personalized approaches in clinical management and treatment.^5^ Second, care standards for hemoglobinopathies remain inconsistent, with substantial gaps between and within countries.^6^ Third, there is limited access to structured training and clinical education, particularly in countries with underdeveloped health systems.^7^ Finally, a fragmented research landscape has impeded the development of innovative, cross‐border strategies that could guide policy and practice.^8^ These limitations are compounded by poor coordination globally, restricting the sharing of knowledge and the development of common standards.

The HELIOS Action (Haemoglobinopathies in European Liaison of Medicine and Science, www.heliosaction.eu) was launched in 2023 to address these persistent gaps, through networking and training activities. Funded by the European Cooperation in Science and Technology (COST), HELIOS is a collaborative open network designed to strengthen research infrastructure, standardize care practices, and promote inclusive participation in hemoglobinopathy‐related science and policy. HELIOS thus provides a coordinated and inclusive platform and facilitates concerted action to tackle major challenges in hemoglobinopathies. Specifically, HELIOS fosters multicentric collaboration and genetic mapping to address the lack of harmonized research, develops regionally adapted clinical protocols to reduce inconsistent standards, expands structured training programs to close education gaps, and strengthens international data sharing and policy engagement to overcome fragmentation. To date, the Action involves over 245 members from 36 countries (Figure 1), and continues to grow, with participants spanning Europe, Africa, Asia, North America, and Australia, reflecting the global relevance of these disorders.

**Figure 1:**
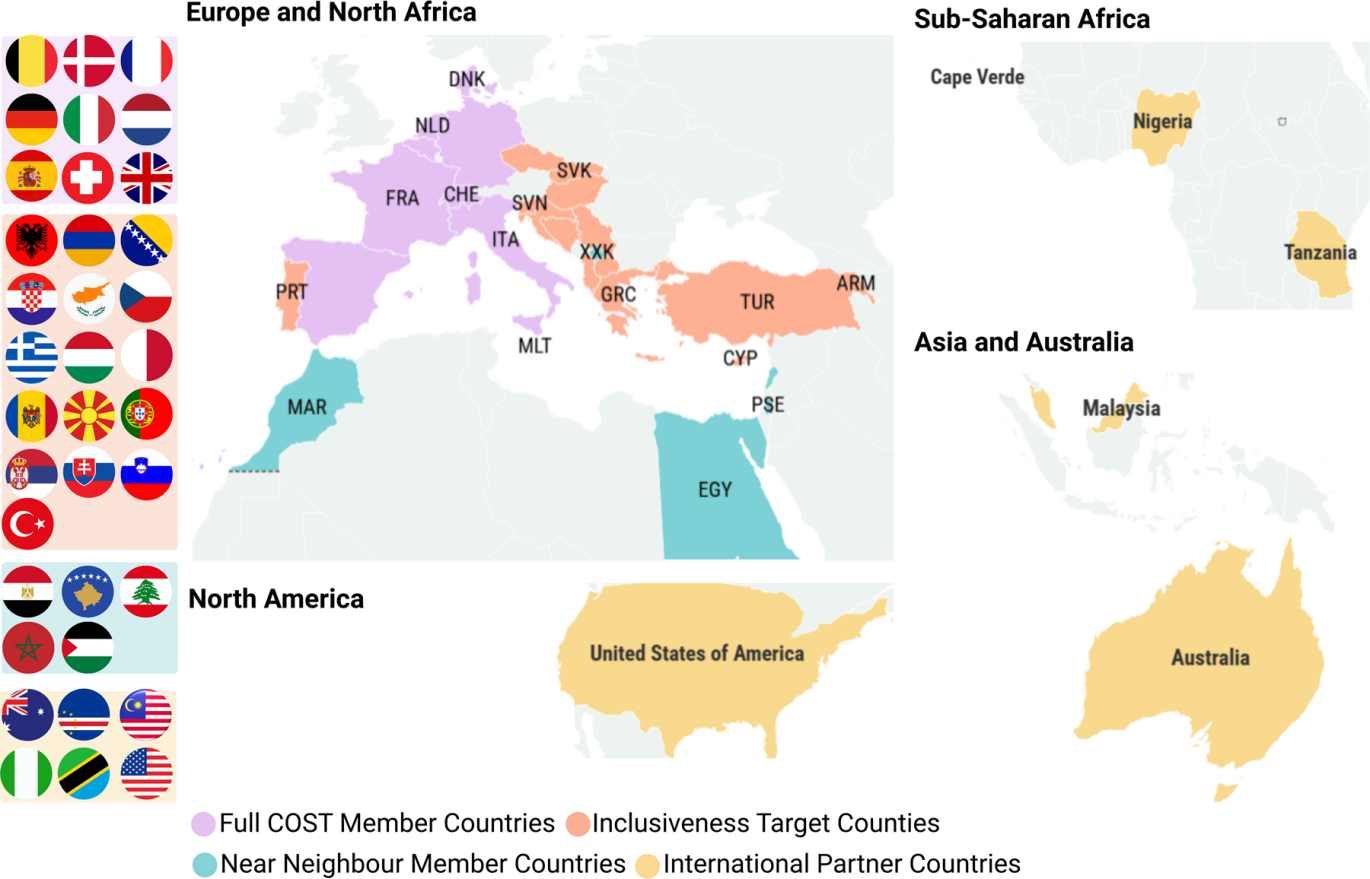
**Global distribution of HELIOS members by country COST membership status**.

A key strength of the HELIOS Action lies in its inclusive and collaborative structure. As a global collaborative program, HELIOS facilitates data standardization, training, policy engagement, and knowledge dissemination, while enabling large‐scale, cross‐border research in hemoglobinopathies through its networking activities. Membership is open to all individuals with an interest in the field, regardless of expertise level. This inclusive approach fosters a model of mutual learning, with participants engaging as trainers, trainees, or both. A central objective of HELIOS is the structured transfer of expertise developed through decades of successful screening, prevention, and clinical management initiatives. Regular working group meetings, annual assemblies, and stakeholder engagement activities ensure coordinated action, broad participation, and sustained momentum across the network.

Of its 245 members so far, about 57% are females, 47% are young researchers and innovators (YRIs, i.e., <40 years old, as defined by the COST association), 53% are senior experts, and 58% are from COST‐designated Inclusiveness Target Countries (ITCs). In the context of COST, ITCs are countries with lower participation in EU research programs, identified to promote geographical balance and inclusiveness in European research. Through HELIOS and other funded Actions, COST supports ITC involvement by providing dedicated funding opportunities and encouraging participation in its open, interdisciplinary research networks. Importantly, HELIOS has established three additional leadership roles: an Ethics, Gender Equality, Diversity Coordinator, a Patient Liaison Coordinator, and a YRIs Coordinator, to ensure equitable access to leadership, networking, and educational opportunities for underrepresented groups. This commitment to diversity reinforces HELIOS' vision of a global and inclusive research network in the hemoglobinopathy field, leaving no region or social group behind.

The timing of this initiative is crucial. Emerging advances in genomic medicine, novel therapies, and digital health tools are transforming the hemoglobinopathy field, making it an ideal moment to enhance knowledge sharing and collaboration through the HELIOS network. With a focus on networking and training activities, HELIOS aims to bridge gaps across disciplines within the hemoglobinopathy field and disseminate knowledge through five interconnected working groups (WGs), with the first three focused on knowledge creation and the remaining two focused on knowledge transfer. The WGs have been deliberately mapped to the four persistent challenges: WG1 and WG3 tackle the lack of harmonized research and data; WG2 promotes consistent clinical standards; WG4 builds capacity through structured training; and WG5 enhances coordination, public awareness, and policy engagement.

1. WG1—Molecular research and diagnosis: focused on diagnostic standardization, genetic mapping, variant interpretation standards, and identifying actionable molecular targets.
2. WG2—Clinical research and patient management: developing regionally appropriate care protocols and assessing therapeutic access.
3. WG3—Data management and interoperability: building registries, developing standardized open data sets, and promoting adherence with the F.A.I.R. (Findable, Accessible, Interoperable, Reusable) data principles.
4. WG4—Education and capacity building: addressing workforce shortages and upskilling health professionals through training and networking activities.
5. WG5—Dissemination and outreach: engaging policymakers, clinicians, patients, and patient advocates, entrepreneurs, and communities with accessible and impactful knowledge products.

HELIOS has prioritized capacity building by implementing diverse training and knowledge exchange initiatives throughout its initial two‐year period. These educational activities engaged over 753 participants, with notable representation including approximately 57% women, 46% YRIs, and 48% participants from ITCs. The specific training programs delivered include the following.

## TRAINING SCHOOLS

- *Training School on molecular research and diagnosis of haemoglobinopathies, Greece, April 2024*: organized by WG1. Participants received training in the diagnosis of hemoglobinopathies and standardized interpretation of globin gene variants, current diagnostic standards, and screening approaches, and applied their knowledge through case‐based, hands‐on sessions.
- *Training School on F.A.I.R. Data Principles, Cyprus, May 2025*: organized by WG3 in collaboration with the EU‐funded HemaFAIR project (no. 101159589. https://hemafairproject.eu/). The program introduced participants to the F.A.I.R. data principles, described methods and tools for making research data F.A.I.R., and included hands‐on training using different data sets relevant to hemoglobinopathies.
- *Training School on Transcranial Doppler, Germany, September 2025*: organized by WG2. This event combined theoretical instruction with practical training on this essential technique for stroke risk assessment in individuals with SCD.

## WEBINAR SERIES (n: NUMBER OF WEBINARS TO DATE)

- *YRI Monthly Discussion Club* (*n* = *6*): Organized by the YRI Coordinator, a series of virtual meetings that offer YRIs a platform to present their work, share ideas and projects, engage with peers, and receive constructive feedback from experts in the field.
- *F.A.I.R. Principles Webinar Series* (*n* = *8*): Organized in collaboration with the HemaFAIR project, the series introduces the F.A.I.R. data principles to the HELIOS network and the broader public through a step‐by‐step approach, using real‐world examples to illustrate their practical application.
- *Clinical WG2 Webinar Series* (*n* = *3*): Organized by WG2, this series covers essential topics in hemoglobinopathy care, including iron overload management, vascular access modalities, and alloimmunization with extensive blood typing. These sessions aim to support clinical best practices and foster knowledge exchange across the network.
- *Invited Speaker Series* (*n* = *8*): A monthly public event that features leading authors of recent publications showcasing the latest advances in hemoglobinopathy research. Topics span diagnosis, clinical care, guidelines, artificial intelligence, patient‐reported outcome measures, and health economics.

The webinars are also available on demand on the HELIOS YouTube Channel (http://www.youtube.com/@heliosaction).

## OTHER TRAINING AND NETWORKING OPPORTUNITIES

HELIOS offers funding support for members to attend high‐level international conferences and participate in short‐term scientific missions (STSMs), enabling direct mentorship and collaboration with experts worldwide. To date, HELIOS has offered 13 conference grants and 12 STSMs. These initiatives foster skill development, strengthen global research networks, and promote knowledge exchange. Furthermore, HELIOS encourages and facilitates collaborative publications among members, contributing to the advancement of the hemoglobinopathy field.

Additionally, HELIOS is developing standardized, disease‐specific surveys and comprehensive needs assessments to systematically map diagnostic gaps, treatment disparities, data availability, relevant policies, and training requirements across participating countries. These tools are important for generating robust data that can inform future research agendas, support the harmonization of clinical and diagnostic protocols, and underpin evidence‐based policy dialogue with health system stakeholders. By anchoring these efforts in a standardized methodology, HELIOS aims to foster consistency, comparability, and cross‐border learning throughout the network.

Recognizing the importance of international and cross‐sectoral alignment, HELIOS has created a dedicated role, an International and Cross‐sectoral Expansion Coordinator, to lead efforts in scaling standardized approaches and deepening engagement with other consortia and sectors. This is pivotal in driving collaboration beyond national boundaries and across disciplines, ensuring that HELIOS is fully integrated into broader rare disease and global health frameworks. Through shared surveys, HELIOS is actively building partnerships with key international networks, including ERN‐EuroBloodNet,^9^ RADeep, INHERENT,^10^ and HemaFAIR. These collaborations promote synergy across European and international research programs, minimize duplication of effort, and align critical resources.

While the HELIOS Action holds promise, it faces challenges, including sustainable funding, reaching diverse populations, and ensuring data interoperability. To address these, HELIOS is actively diversifying funding sources, engaging local communities, and establishing clear data standards, aiming at maximizing its impact on hemoglobinopathy research and care.

The growing global burden of hemoglobinopathies, intensified by migration and demographic transitions, demands coordinated international action. Through its commitment to standardization, equity, and cross‐border collaboration, HELIOS is building the infrastructure and partnerships needed to help shape the future of hemoglobinopathy research, policy, and care within Europe and beyond. By aligning its activities with the key unmet needs, that is, multicentric research, harmonized standards, structured training, and coordinated action, HELIOS offers a timely and convincing framework to overcome the persistent hurdles in sickle cell disease and thalassemia care.

## AI USE DISCLOSURE

During the preparation of this work, the authors used ChatGPT in order to improve the readability and language of the work. After using this tool, the authors reviewed and edited the content as needed and take full responsibility for the content of the publication.

## AUTHOR CONTRIBUTIONS

**Sotiroula Chatzimatthaiou**: Conceptualization; methodology; data curation; writing—original draft; writing—review and editing; visualization; project administration; validation; formal analysis. **Fedele Bonifazi**: Writing—review and editing; validation. **Anna C. Gimbert**: Writing—review and editing; validation. **Raffaella Colombatti**: Writing—review and editing; validation. **Francesco Cremonesi**: Writing—review and editing; validation. **Andreas Glenthøj**: Writing—review and editing; validation. **Elisabetta Mezzalira**: Writing—review and editing; validation. **Coralea Stephanou**: Writing—review and editing; validation. **Jan Traeger‐Synodinos**: Writing—review and editing; validation. **Darko Antic**: Writing—review and editing; validation. **Burak Durmaz**: Writing—review and editing; validation. **Eleni Gavriilaki**: Writing—review and editing; validation. **Baba Inusa**: Writing—review and editing; validation. **Annalisa Landi**: Writing—review and editing; validation. **Mariangela Pellegrini**: Writing—review and editing; validation. **Francesca Basile**: Writing—review and editing; validation. **Stephanie Muth**: Writing—review and editing; validation. **Holger Cario**: Writing—review and editing; validation. **Eleni Katsantoni**: Writing—review and editing; validation. **Dilem Ruhluel**: Writing—review and editing; validation. **Carsten W. Lederer**: Writing—review and editing; validation. **María del Mar Mañú‐Pereira**: Writing—review and editing; validation. **Petros Kountouris**: Writing—review and editing; writing—original draft; conceptualization; supervision; project administration; visualization; validation; funding acquisition.

## CONFLICT OF INTEREST STATEMENT

A.G.: Grants or contracts from: Novo Nordisk, Sanofi, Bristol Myers Squibb, Agios Pharmaceutical (to the institution); Consulting fees: Novo Nordisk, Pharmacosmos, Disk Medicine (personal); Data Safety Monitoring Board/Advisory Board: Disc Medicine, Vertex Pharmaceuticals, Novo Nordisk. BPDI; Support for the present manuscript: Novo Nordisk (personal/employment); Stock or stock options: Novo Nordisk (personal/employment benefits). C.W.L.: Support for the present manuscript: COST CA22119 (to the institution), CING (personal); Grants or contracts from: COST CA22119 (personal and to the institution). H.C.: Consulting fees: BMS, Pfizer (personal); Payment or honoraria: Chiesi, Pfizer, Vertex, ApoCare (personal); Support for attending meeting(s) and/or travel: Pfizer; Participation on a Data Safety Monitoring Board or Advisory Board: Pfizer, Agios (personal). M.M.P.: Grants or contracts from: Agios Pharmaceuticals, BMS, Novo Nordisk, Novartis Pharma, Pfizer (institutional). P.K.: Grants or contracts from: Agios Pharmaceuticals (institutional).

## ETHICS STATEMENT

Ethical approval was not sought for this article because it does not contain any studies involving human participants performed by any of the authors.

## FUNDING

This article/publication is based upon work from COST Action HELIOS, CA22119, supported by COST (European Cooperation in Science and Technology).

## Data Availability

Data are publicly available on HELIOS' Zenodo account (https://zenodo.org/communities/ca22119).
